# Neurologist’s Black Swan: Molecular Basis of Prenatal Seizures

**DOI:** 10.3390/ijms27010283

**Published:** 2025-12-26

**Authors:** Angelina O. Kustova, Alexandra D. Medyanik, Polina E. Anisimova, Victor S. Tarabykin, Elena V. Kondakova

**Affiliations:** 1 Institute of Neuroscience, Lobachevsky State University of Nizhny Novgorod, 23 Gagarin Ave., 603022 Nizhny Novgorod, Russia; elakust@gmail.com (A.O.K.); al.medyanik111@gmail.com (A.D.M.); polina.adyasova@yandex.ru (P.E.A.);; 2 Department of Genetics and Life Sciences, Sirius University of Science and Technology, 1 Olympic Ave, 354340 Sochi, Russia

**Keywords:** fetal seizures, epilepsy, developmental and epileptic encephalopathies, pathogenic variant, voltage-gated sodium channels

## Abstract

One of the least studied but clinically severe forms of epilepsy is seizures with prenatal manifestations. Our understanding of epilepsy disorders has advanced substantially; numerous disease-associated genes have been identified, classifications have been refined, and underlying mechanisms and diagnostic approaches have been elucidated. However, one group of patients—those with seizures before the birth—has remained largely overlooked by researchers, despite numerous similar clinical cases reported over the past two decades. To date, only two genes, *SCN2A* and *SCN8A*, have been shown to have pathogenic variants that are reliably related to fetal epilepsy. Yet, how many genes are truly involved? This review will examine the known molecular foundations of epilepsy with prenatal onset. The prevalence of fetal seizures in patients with epilepsy is likely underestimated, although timely diagnosis of the disease is crucial for patient outcomes.

## 1. Introduction

Historically, fetal seizures were considered to be associated primarily with external factors such as infections, exposure to toxins, or metabolic abnormalities [1]. As for genetic reasons, prenatal onset of epilepsy is uncharted territory [2,3,4,5,6,7]. The limited availability of non-invasive prenatal diagnostic techniques makes early detection of seizures particularly challenging. Routine ultrasonography, even when performed during a suspected episode, has low sensitivity and specificity for detecting seizures in utero. Emerging non-invasive techniques, such as fetal magnetoencephalography, remain technically challenging and are available only in a limited number of centers. Maternal perception is inherently subjective and may reflect either epileptic activity or benign fetal movements such as hiccups, turns, or sleep-related movements [8,9].

As a result, fetal seizure diagnosis is often retrospective and inferential, relying on indirect indicators such as stereotypy and recurrence of movements, their persistence across fetal behavioral states, and subsequent postnatal confirmation of epilepsy or structural brain abnormalities. The absence of validated screening and verification tools contributes to substantial underdiagnosis, delaying appropriate clinical management and limiting opportunities for prenatal intervention. In this context, identifying reliable genetic markers associated with fetal seizures becomes particularly important.

To date, there are many known cases of fetal epilepsy. Unfortunately, genetic analyses have not been performed in most instances. The first such clinical case was published in 1996 [10]. At 32 weeks of gestation, during an ultrasound examination, clinicians observed idiopathic polyhydramnios and fetal seizures characterized by rapid, repetitive limb movements lasting 30–60 s and occurring on multiple occasions. After birth, the infant continued to exhibit tonic–clonic seizure activity [10]. More recent data also indicate severe outcomes in patients with prenatal seizure onset [8,11,12,13]. According to available statistics, more than 50% of documented cases were associated with developmental anomalies of the central nervous system [14]. Structural brain abnormalities, including cortical damage or disorganization, may represent either primary developmental defects or secondary consequences of recurrent epileptic activity after birth [15]. Early detection of such conditions enables timely initiation of anticonvulsant therapy and may improve patient prognosis [3]. However, timely diagnosis and appropriate treatment of prenatal seizures are not feasible without an understanding of the molecular pathways involved. In our review, we focused on genes that have a proven link to prenatal seizures. According to the results of DNA sequencing of patients with recorded seizures during embryonic development, only two genes have been identified: *SCN2A* and *SCN8A*. Both encoding α-subunits of voltage-gated sodium channels (NaV) [2,3].

There are four main subtypes of voltage-gated sodium channels (VGSCs) in the human central nervous system: NaV1.1, NaV1.2, NaV1.3, and NaV1.6, encoded by the *SCN1A*, *SCN2A*, *SCN3A*, and *SCN8A* genes, respectively [5]. During embryonic and postnatal development, the expression of these genes, the cellular localization of their protein products, the diversity of isoforms, and even their functional properties undergo dynamic and finely tuned changes. These developmental dynamics underscore the critical role of these genes in the formation and maturation of the nervous system during early ontogenesis [16,17,18,19,20]. Pathogenic variants in the *SCNxA* gene family have been implicated in a broad spectrum of epileptic and neurodevelopmental disorders (NDDs), ranging from severe developmental and epileptic encephalopathy (DEE) to milder forms of epilepsy, autism spectrum disorders (ASD), and intellectual disability [21,22].

**Figure 1:**
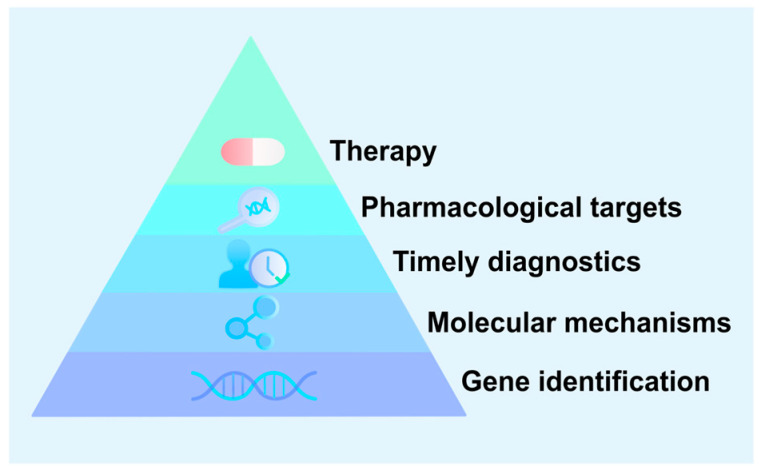
From genes to therapy: a translational pyramid. The identification of new genes underlies the development of diagnostic and therapeutic approaches for congenital forms of epilepsy.

The research community should focus more closely on populations at risk for prenatal seizures, such as families with children affected by early-onset epilepsy or congenital central nervous system malformations [23]. To develop effective diagnostic and therapeutic protocols for prenatal epilepsy, it is essential to identify pathogenic variants in affected individuals and elucidate the molecular genetic mechanisms that trigger seizure activity (Figure 1).

## 2. SCN2A

### 2.1. SCN2A Patients with Prenatal Seizures

SCN2A-associated disorders represent a heterogeneous group of monogenic diseases of the central nervous system caused by pathogenic variants in the *SCN2A* gene [24]. The clinical phenotype encompasses a broad spectrum of neurological manifestations, ranging from catastrophic early infantile epileptic encephalopathies (EIEE11, OMIM #613721) [25,26] to autism spectrum disorders and intellectual disability without an epileptic component [27]. SCN2A-associated disorders are typically not accompanied by structural brain abnormalities [22], although the cases described below represent notable exceptions.

Sauvestre F. et al. reported a clinical case of prenatal seizures caused by a *de novo* missense variant in the *SCN2A* gene [2]. The pregnant woman underwent regular monitoring, and an ultrasound examination at 20 weeks of gestation revealed no abnormalities. However, at 30 weeks, a routine scan detected multiple structural abnormalities and repetitive fetal tonic–clonic movements. Medical termination of pregnancy was performed at 32 weeks. Whole-genome sequencing identified a missense variant in *SCN2A* (c.4471A>G, p.Ile1491Val). Examination revealed fetal akinesia and developmental abnormalities of the corpus callosum, dentate nuclei, and olivary nuclei. Heterotopic calcified neuronal clusters were observed in the intermediate zone of the cerebral hemispheres, along with scattered calcified neurons in the striatum. No cortical dysplasia or hippocampal lesions were detected [2].

Additional reports describe congenital structural brain abnormalities associated with pathogenic *SCN2A* variants. In both cases, status epilepticus occurred in the neonatal period, although prenatal onset of epileptic activity was suspected [4,5].

Bernardo S. reported a case of severe epileptic encephalopathy accompanied by extensive cortical dysplasia [5]. At 31 weeks of gestation, fetal ultrasound revealed bilateral ventriculomegaly, which was confirmed by MRI at 32 weeks. Further imaging indicated pachygyria, corpus callosum dysgenesis, absence of cortical-white matter differentiation, and signs of neuronal disorganization. The mother had no significant family history, no evidence of infection, and both fetal karyotyping and array-CGH were normal. Labor was induced at 34 weeks due to fetal tachycardia. On the sixth day of life, the newborn developed focal status epilepticus with associated bradycardia and oxygen desaturation, partially controlled with antiepileptic drugs. Postnatal MRI confirmed previously noted abnormalities and revealed additional foci of ischemia in the frontoparietal regions. Exome sequencing identified a *de novo SCN2A* variant (c.751G>A, p.Val251Ile). The authors emphasized the importance of molecular genetic diagnosis for identifying severe structural brain abnormalities in utero, even when family history and standard genetic testing yield normal results [5].

A cohort study by Hadjipanteli et al. (2024) [4] described a fetus presenting with severe prenatal abnormalities, including ventriculomegaly, clenched hands, and intrauterine growth restriction, detected via prenatal ultrasound. Karyotyping and array-CGH revealed no abnormalities, and only trio-exome sequencing detected a *de novo* heterozygous missense *SCN2A* variant (c.751G>A, p.Val251Ile). After birth, the child presented with epileptic spasms, skeletal muscle atrophy, and refractory status epilepticus. MRI revealed severe cortical abnormalities, including polymicrogyria and lissencephaly. Despite intensive treatment, the patient died in the neonatal period [4].

The cases described above share a distinctive feature that sets them apart from most SCN2A-associated disorders: the presence of structural brain abnormalities. Notably, the same *de novo* missense variant (c.751G>A, p.Val251Ile) in the *SCN2A* gene was identified in multiple observations. The repeated detection of this mutation in patients with severe congenital brain malformations and epileptic encephalopathy emphasizes its clinical relevance [4,5].

The identification of new pathogenic variants, particularly *de novo* mutations, not only expands our understanding of the phenotypic spectrum of SCN2A-associated disorders but also enhances the quality of molecular genetic diagnostics. Including such variants in diagnostic panels enables faster, more accessible, and more cost-effective detection in new patients, which is critical for early diagnosis and timely initiation of therapy [2,5]. SCN2A-related pathologies arise from a wide range of mechanisms, including the functional nature of the mutation, age-dependent alternative splicing, interactions with other ion channels, and the influence of regulatory proteins. Each mechanism involves distinct molecular pathways that contribute to the phenotypic heterogeneity of SCN2A-associated diseases and necessitate individualized therapeutic approaches. Therefore, in addition to identifying new pathogenic variants, elucidating the molecular mechanisms in which the pathogenic protein is involved is essential.

### 2.2. Molecular Basis of SCN2A-Associated Epilepsy

The *SCN2A* gene encodes the α-subunit of the voltage-gated sodium channel subtype NaV1.2 [28]. This channel is widely expressed in the central nervous system, predominantly in glutamatergic pyramidal neurons. The highest density of NaV1.2 is found in the axon initial segment (AIS), although it is also present in the soma and dendrites [18,29,30,31,32]. The α-subunit forms the core structural component of the channel and determines its key electrophysiological properties, including ion conductance and activation/inactivation kinetics [22,30,33].

**Figure 2:**
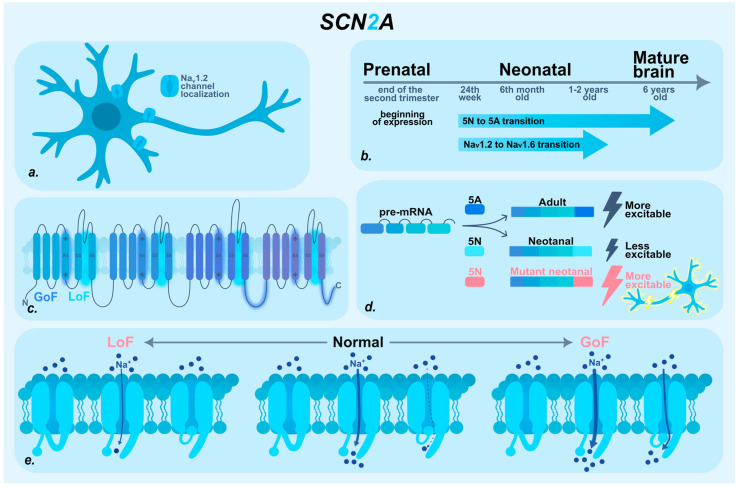
*SCN2A* in fetal neurodevelopment: localization, temporal expression and pathogenic mechanisms. (**a**) Subcellular localization of Nav1.2 in neurons. Nav1.2 is expressed in the axon initial segment, soma and dendrites of pyramidal neurons. (**b**) Timeline of *SCN2A* expression and transitions during prenatal and neonatal period. (**c**) Structural map of the Nav1.2 channel. LoF variants localized in the pore-forming S5-S6 segments. GoF variants enriched in domains critical for inactivation: S4 voltage sensor, DIII–DIV linker, and the C-terminal region. (**d**) Age-dependent alternative splicing of exon 5. The adult 5A isoform is more excitatory, whereas the neonatal 5N isoform reduces excitability to protect the developing nervous system. However, pathogenic variants in 5N can disrupt this balance and induce abnormal excitability. (**e**) Functional consequences of GoF and LoF *SCN2A* variants on NaV1.2 channel behavior. GoF variants impair inactivation, producing a window Na^+^ current that drives hyperexcitability. LoF variants reduce conductance and current density, lowering neuronal excitability.

The heterogeneity of SCN2A-associated phenotypes can be attributed to multiple factors, including developmental regulation of gene expression through alternative splicing, the nature of the underlying mutation, and the influence of modifier proteins [27,34,35,36,37] (Figure 2).

NaV1.2 expression begins prenatally at the end of the second trimester [38]. During the neonatal and early postnatal periods, NaV1.2 is the predominant sodium channel, fully mediating the initiation and propagation of action potentials and participating in synaptic transmission and dendritic function [18,39,40]. After 1–2 years of age, NaV1.2 is partially replaced: it continues to mediate action potential propagation into the somatodendritic compartments of neurons, but the primary role in action potential initiation shifts to the NaV1.6 channel [18,29,38,41].

The functional regulation of *SCN2A* is significantly influenced by age-dependent alternative splicing of exon 5, which exists in two mutually exclusive variants: the neonatal (5N) and adult (5A) isoforms. These isoforms differ by only a single amino acid—5N contains asparagine, whereas 5A contains aspartic acid. Despite this minor difference, the substitution occurs within the voltage sensor domain, markedly impacting sensitivity to changes in membrane potential [20,27,42,43,44]. The transition from the neonatal to the adult isoform begins around 24 weeks of gestation and continues actively during the first six months of life, with some brain regions transitioning up to six years of age [27,45]. Under physiological conditions, the neonatal 5N isoform is less excitable, as it activates at a higher membrane potential, thereby reducing neuronal excitability in the developing brain. However, in the presence of a pathogenic variant in the 5N isoform, this balance is altered: shifts in voltage-dependent inactivation lead to the formation of a window current, membrane depolarization, and resultant neuronal hyperexcitability. Under these conditions, even a moderate increase in channel current can significantly disrupt the excitation–inhibition balance, favoring hyperexcitability and contributing to early disease onset [16,42,44,45,46,47].

Regarding the nature of the mutation, researchers traditionally distinguish two primary functional categories of mutant channels: gain-of-function (GoF) and loss-of-function (LoF). Typically, gain-of-function variants are associated with early-onset epileptic disorders, whereas loss-of-function variants are linked to late-onset epilepsy and autism spectrum disorders [45,48,49]. However, emerging evidence indicates that this binary classification is overly simplistic. Among variants associated with early-onset epileptic disorders studied by Thompson et al., a range of functional effects was observed, including classical GoF, mixed dysfunction, and clear LoF phenotypes [34].

LoF variants of *SCN2A* are most commonly located within the channel pore region (segments S5-S6), which forms the ion-conducting pathway. Such variants impair conductivity and reduce ion current density, leading to decreased neuronal excitability. During early development, this reduction in excitability can significantly disrupt the formation of neural networks, potentially contributing to cognitive impairment. Clinically, LoF variants are generally associated with milder phenotypes, including ASD, NDDs without epilepsy, or late-onset seizures [49,50]. However, in some cases, LoF variants alter NaV1.2 inactivation kinetics, which has been linked to the development of DEE [51].

However, it is important to keep in mind that in the case of *de novo* mutations, a dominant-negative (DN) effect may phenotypically resemble a LoF, but the underlying mechanism is different. A DN protein might interfere with the function of the wild-type protein. As a result, the cell loses the normal function of the protein, and the phenotype appears as a classic LoF. Nevertheless, the function is not lost due to the absence of the protein but because its activity is suppressed by the mutant form, which is crucial for therapeutic development [52,53]. Such mechanisms have not yet been identified for the *SCNxA* gene family, but *de novo* missense variants with DN effects do occur in epilepsy, for example, in genes such as *STXBP1, KCNQ2, KCNQ3,* and *SMC1A* [54,55,56,57]. It is important to consider the possibility of such variants during diagnostics.

GoF variants of *SCN2A* are more frequently localized in regions critical for channel inactivation, such as the voltage sensor domain (S4), the linker between domains III and IV, and the C-terminal region of the protein. These variants disrupt normal inactivation mechanisms—either by slowing inactivation, speeding recovery from inactivation, or causing partial inactivation failure. This results in the formation of a persistent or “window” current of Na^+^ ions, promoting membrane depolarization and leading to neuronal hyperexcitability [28,34,58]. In the mature brain, the role of NaV1.2 in action potential initiation decreases, which mitigates the impact of GoF variants [16,17,29]. However, during early development—when NaV1.2 is the predominant sodium channel in excitatory neuron axons and the inhibitory system remains depolarizing—such pathogenic variants can have severe consequences, disrupting neuronal activity even before birth. This makes GoF SCN2A variants plausible candidates for the molecular basis of prenatal seizures. Nevertheless, individual variants may affect multiple biophysical properties of the channel simultaneously, combining features of both GoF and LoF and thereby complicating prediction of their clinical consequences.

In addition to pathogenic changes in channel structure and alternative splicing, intracellular modulator proteins play an important role in regulating NaV1.2 function. For example, the intracellular fibroblast growth factor (iFGF) subfamily, including FGF11–FGF14, directly interacts with NaV channel α-subunits. These proteins regulate channel gating properties and membrane density, thereby influencing neuronal excitability [36,59,60,61,62,63]. Another key regulator is calcium/calmodulin-dependent protein kinase II (*CaMKII*). Experiments with mutant NaV1.2 demonstrated that *CaMKII* activity enhances persistent sodium current and shifts inactivation to more depolarized membrane potentials, prolonging channel activity and increasing neuronal excitability. In contrast, inhibition of *CaMKII* eliminated these effects: persistent current was abolished, inactivation increased, and the frequency of spontaneous action potentials decreased [37]. Thus, both iFGFs and *CaMKII* provide additional regulatory layers capable of modulating NaV1.2 function during development. In the presence of an *SCN2A* mutation, these modulators may amplify pathogenic effects, shifting a moderate functional change toward pronounced hyperexcitability, particularly during critical stages of early development. This multilevel interaction increases the likelihood that even modest functional alterations may lead to clinically significant manifestations, including prenatal seizure onset.

The severity of the phenotype may also be influenced by the patient’s genetic background. For example, in an Scn2a mouse model, modifier alleles significantly affected disease severity [64].

In summary, the pathogenesis of *SCN2A* variants is determined by the interplay of several mechanisms: the nature and location of the mutation, interactions with other channels (e.g., NaV1.6), age-dependent alternative splicing of exon 5, and the influence of modulator proteins. Each factor alters NaV1.2 kinetics in distinct ways. Their combined effects become particularly critical during early ontogenesis, when even moderate functional disruptions can manifest as severe phenotypes, including seizures in the neonatal or even prenatal period.

The diversity of molecular mechanisms underlying SCN2A-related disorders underscores the importance of variant identification. To accurately interpret the clinical phenotype and select appropriate therapy, it is crucial to consider not only the nature of the mutation itself but also its broader molecular and developmental context.

## 3. SCN8A

### 3.1. SCN8A Patients with Prenatal Seizures

Pathogenic variants in the *SCN8A* gene are associated with early infantile epileptic encephalopathy type 13 (EIEE13; OMIM #614558) [25], a disorder characterized by pronounced phenotypic heterogeneity and a severe clinical course [3,65]. The disease typically begins at 4–5 months of age, presenting with multiple seizure types that rapidly become refractory to treatment [66]. Patients with EIEE13 commonly exhibit psychomotor delay, intellectual disability, hypotonia, dystonia, hyperreflexia, and ataxia [67,68]. Prenatal clinical or subclinical seizures are likely more common in this pathology than currently recognized and may serve as an early indicator of more severe neurological impairment. The overall prognosis is unfavorable and often accompanied by severe movement disorders [6,7].

One of the earliest reported links between prenatal seizures and a pathogenic *SCN8A* variant was described by Singh et al. (2015) [6]. The proband was born at 40 weeks of gestation with no signs of dysmorphic anomalies. The mother reported unusual rhythmic fetal movements in late pregnancy, despite normal results from antenatal examinations. After birth, the infant demonstrated jittery limb movements and a pronounced startle response to tactile and acoustic stimuli, mimicking hyperekplexia. At just 15 h old, the first clonic seizure occurred, necessitating hospitalization. Over time, frequent stereotypical tonic and tonic–clonic seizures developed, refractory to anticonvulsant treatment, while jittery movements persisted and were exacerbated by stress. Brain MRI in the early neonatal period was normal, but subsequent imaging revealed cerebellar volume loss. Genetic testing identified a *de novo* SCN8A mutation (c.3979A>G; p.Ile1327Val) [6].

McNally et al. (2016) [3] presented a clinical observation highlighting seizure treatment in the context of prenatal onset, underscoring the importance of early detection and therapeutic intervention. At 26 weeks of gestation, ultrasound revealed unusual repetitive “convulsive” fetal movements, accompanied by generalized fetal edema (anasarca), joint contractures (arthrogryposis), macrosomia, reduced motor activity, and limb posture abnormalities. Behavioral assessment by ultrasound [69] indicated signs of global cerebral hyperexcitability. Anticonvulsant therapy resulted in a reduction in seizure-like movements. The infant was born with marked edema, contractures of the limbs, craniofacial anomalies, and clenched fists with overlapping fingers. Postnatal evaluation confirmed neurological impairment and structural cerebellar anomalies. Whole exome sequencing revealed a *de novo SCN8A* mutation (c.718A>G; p.Ile240Val) [3].

Interestingly, Denis et al. (2019) also reported similar abnormal fetal movements in the third trimester, with two mothers among 19 cases with SCN8A mutations noting signs suggestive of intrauterine seizure activity [7].

Thus, *SCN8A* variants may manifest prenatally as atypical fetal movements, which should raise concern among obstetricians monitoring pregnancies with similar reports. Understanding the molecular mechanisms underlying SCN8A-associated disorders not only allows for more accurate prognosis and individualized therapy but also facilitates the development of targeted treatments. Molecular genetic diagnosis of pathogenic *SCN8A* variants may therefore play a critical role in modern clinical practice for patients with suspected DEE.

### 3.2. Molecular Basis of SCN8A-Associated Epilepsy

The *SCN8A* gene encodes the α-subunit of the voltage-gated sodium channel subtype NaV1.6. This channel is widely expressed in both excitatory and inhibitory neurons of the central and peripheral nervous systems. Functionally, NaV1.6 plays a pivotal role in action potential initiation, gradually replacing NaV1.6 with age. It is also responsible for regulating nerve impulse conduction velocity [29,41,70,71,72,73,74,75].

**Figure 3:**
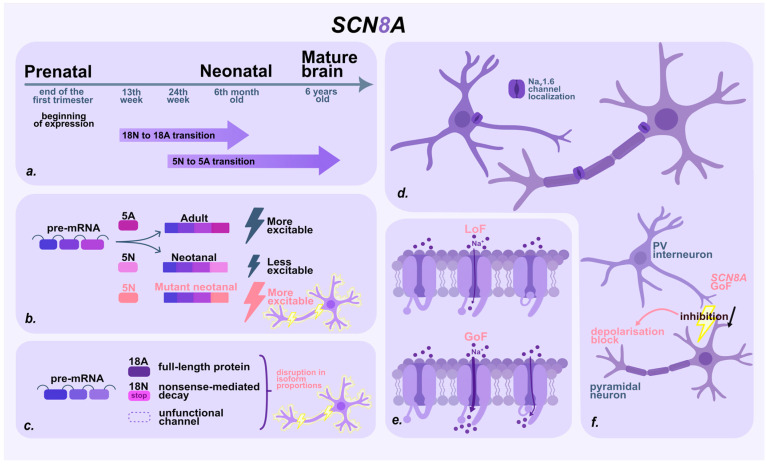
*SCN8A* in fetal neurodevelopment: localization, temporal expression and pathogenic mechanisms. (**a**) Timeline of *SCN8A* expression and transitions during prenatal and neonatal period. (**b**) Age-dependent alternative splicing of exon 5. The adult 5A isoform is more excitatory, whereas the neonatal 5N isoform reduces excitability to protect the developing nervous system. However, pathogenic variants in 5N can disrupt this balance and induce abnormal excitability. (**c**) Age-dependent alternative splicing of exon 18. Prenatally, most transcripts either include exon 18N or skip exon 18 altogether, producing nonfunctional channels. Later, a switch occurs to exon 18A. An imbalance in the ratio of these isoforms can cause neuronal hyperexcitability. (**d**) Subcellular localization of Nav1.2 in neurons. Nav1.6 is localized in both excitatory and inhibitory neurons, at the distal part of the axon initial segment and at the nodes of Ranvier in myelinated axons. (**e**) Functional consequences of GoF and LoF *SCN2A* variants on NaV1.2 channel behavior. GoF variants impair inactivation (slowed inactivation, faster recovery, or partial loss), producing a persistent/window Na^+^ current that drives hyperexcitability. LoF variants reduce conductance and current density, lowering neuronal excitability; (**f**) GoF *SCN8A* variants in PV interneurons reduce inhibitory output. Membrane hyperexcitability can cause depolarization block, impairing their ability to restrain pyramidal neurons and promoting hyperexcitability.

The mechanisms underlying SCN8A-associated disorders share many similarities with those described for *SCN2A*. However, differences in temporal expression patterns, intracellular channel localization, and specific regulatory mechanisms contribute to a distinct spectrum of clinical phenotypes. As with *SCN2A*, the clinical manifestation of SCN8A-related disorders depends on the nature of the mutation, age-dependent alternative splicing, regulatory protein interactions, and the functional impact of the variant [27,74,76,77,78] (Figure 3).

The timing of *SCN8A* expression in humans remains poorly characterized, but expression levels are generally low during early fetal development and increase approximately two months before birth. Age-dependent splicing has also been shown to begin as early as the 13th week of gestation [27].

Unlike *SCN2A, SCN8A* undergoes age-dependent alternative splicing not only of exon 5 but also of exon 18 [27,79]. Inclusion of exon 18A results in the synthesis of a functional protein, whereas the inclusion of exon 18N produces mRNA containing a premature stop codon, leading to its degradation via nonsense-mediated decay [27,80]. The transition to inclusion of exon 18A in the transcript—and thus the synthesis of full-length NaV1.6—begins gradually at approximately the 13th week of gestation and is completed by about six months after birth. In the early stages of development, the majority of transcripts either contain exon 18N or skip exon 18 entirely, resulting in the production of nonfunctional channels and reduced neuronal excitability [27,58,80]. Notably, the timing of the 18A/18N switch begins and ends significantly earlier than the 5N/5A transition and similar age-dependent splicing events in other sodium channel genes. Splicing of exon 18 is thus a distinctive mechanism underlying early NaV1.6-associated pathologies [27].

Analysis of the temporal dynamics of *SCN8A* splicing isoforms provides a possible explanation for why pathogenic variants in this gene may lead to prenatal seizures. Early in development, nonfunctional transcripts containing exon 18N predominate in the brain, together with the less excitable 5N isoform. An imbalance in the isoform ratio may lead to neuronal hyperexcitability even during the prenatal period.

As with SCN2A, the broad spectrum of clinical manifestations of pathogenic *SCN8A* variants is associated with the functional effect of the mutation—either GoF or LoF. GoF variants are more frequently identified and are associated with severe epilepsy and early-onset DEE [71,72,76,81,82]. These variants facilitate channel activation, impair inactivation, and increase persistent sodium current, resulting in excessive and prolonged influx of Na^+^ ions, a lowered action potential threshold, and ultimately neuronal hyperexcitability [81,83,84,85]. In contrast, LoF variants are associated with milder phenotypes, including cognitive and motor impairments and autism. These disorders arise from decreases in sodium current and disruptions in the excitation pattern [81,84,85,86].

Prenatal seizures are likely more often associated with GoF variants in excitatory neurons, due to increased sodium current and impaired channel inactivation. However, other mechanisms may also contribute, including effects on inhibitory neurons. NaV1.6 channels are highly expressed in parvalbumin-positive (PV) interneurons [78,87,88,89]. Paradoxically, GoF variants of *SCN8A* in these cells can reduce inhibitory activity. Membrane hyperexcitability may lead to depolarization block, a condition in which the neuron ceases to generate action potentials. As a result, the capacity of these inhibitory neurons to suppress pyramidal cell activity is reduced, promoting network hyperexcitability that can trigger seizures and increase the risk of sudden unexpected death in epilepsy (SUDEP) [78,87,89].

In addition, NaV1.6 channel function is regulated by intracellular proteins that influence channel kinetics and localization. Pathogenic variants in genes encoding regulatory proteins can lead to similar phenotypes. Moreover, interactions between these proteins and *SCN8A* variants may exacerbate the clinical presentation [36,58,77]. These interactions should therefore be considered when evaluating patients.

**Figure 4:**
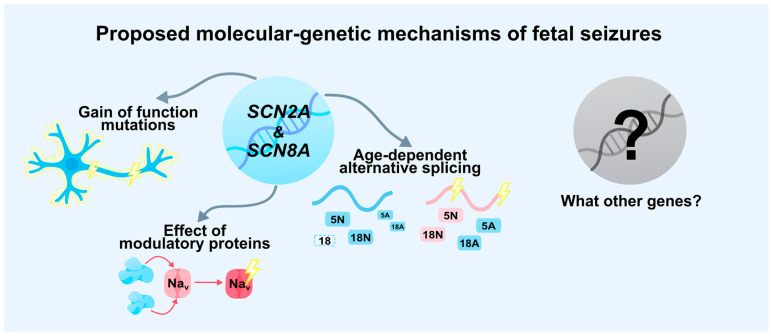
Proposed molecular genetic mechanism of fetal seizures. The known genes *SCN2A* and *SCN8A* can lead to neonatal and potentially prenatal seizures through GoF mutations, shifts in isoform ratios in age-dependent alternative splicing. and the influence of modulatory proteins. Yet it remains unclear how many additional genes and mechanisms of this kind are still undiscovered.

In summary, the interplay between mutation type, alternative splicing, and regulatory proteins disrupts the delicate balance of NaV1.6 expression and function during critical stages of brain development. As with *SCN2A*, pathogenic *SCN8A* variants alter sodium current kinetics and perturb neuronal excitability. However, unique features—such as the temporal expression profile of *SCN8A*, the distinct splicing pattern of exon 18, and channel distribution in both excitatory and inhibitory neurons—distinguish its pathogenic mechanisms from those of other genes. The emergence of GoF variants during early ontogenesis, particularly those affecting the proportion of neonatal and adult channel isoforms, may lead to hyperexcitability and precipitate seizures as early as the prenatal period (Figure 4).

## 4. Discussion

Despite its potentially high prevalence and severe consequences, fetal epilepsy remains a “terra incognita” in modern neurology. A major issue lies in its regulatory and classification ambiguity. The current ILAE classification of epilepsies does not include the formal concept of fetal epilepsy [90]. As a result, seizures that begin in utero are often either unrecognized or incorrectly classified as neonatal onset, distorting epidemiological data and complicating efforts to investigate their specific causes.

This conceptual gap inevitably contributes to a lack of attention to the problem. Diagnosis often relies on maternal subjective reports of abnormal fetal movements (e.g., intense rhythmic twitching or apparent respiratory pauses), which are difficult to verify in the absence of advanced prenatal EEG monitoring, a technique that is not routinely available in clinical practice. Consequently, fetal epilepsy remains largely marginalized in both scientific research and clinical care.

This leads to a key premise: a significant proportion of early neonatal epilepsy cases—particularly those presenting within the first 24–48 h of life—may in fact be prenatal in origin. These infants are born with an already active epileptic process. Studies of EEG patterns in newborns with congenital brain malformations clearly demonstrate that epileptiform activity often begins well before birth [91]. When we encounter a neonate with drug-resistant seizures and a structural brain abnormality (such as focal cortical dysplasia), there is a probability that we are observing the consequences of a process initiated prenatally.

The analysis conducted indicates that at present, only two genes, *SCN2A* and *SCN8A*, have genetic evidence supporting their role in the development of seizures occurring during intrauterine life. Within the scope of this review, we have identified potential candidate genes in which pathogenic variants have been detected in patients presenting with the earliest neonatal seizures: *SCN1A*, *KCNQ2*, *KCNQ3*, *KCNT1*, *STXBP1*, *ARX*, *SLC25A22*, *SLC12A5*, and *ALDH7A1* (Table 1). These genes are expressed during prenatal brain development according to publications and data from the Allen Brain Atlas [27,92,93,94,95,96]. It allows us to hypothesize that the most severe and functionally significant variants in these genes may shift the onset of epileptic seizures to the prenatal period. These genes are proposed for further investigation in fetal seizure etiologies.

In this context, in-depth investigation of genetic causes becomes not only preferable but the most reliable pathway to resolving the current diagnostic limitations. Genetic findings can serve as objective markers confirming prenatal onset (Figure 4).

A major insight of recent years is the recognition that “epilepsy genes” are often neurodevelopmental genes. Mutations in genes *STXBP6*, *CUX2*, *ACTL6B*, *CNTNAP2*, etc. disrupt neuronal migration, cortical organization, and synaptogenesis [110]. A study by Bahi-Buisson et al. [111] demonstrated how mutations in the TUBA1A gene lead to cortical malformations such as lissencephaly and microlissencephaly, frequently accompanied by prenatal seizures and severe epilepsy. Thus, the identification of such a variant in a newborn with seizures on the first day of life could be evidence of prenatal origin. Moreover, this shifts the focus from treating seizures as a symptom to addressing the underlying neurodevelopmental disorder.

Interestingly, prenatal expression of the 5N isoforms is most pronounced for *SCN2A* and *SCN8A* compared to other genes in their family [27], which correlates with the clinical association of pathogenic variants in these genes with prenatal seizure onset. Although previously associated primarily with postnatal epileptic encephalopathies, these genes are now increasingly identified in cases with fetal-onset seizures, prompting a reconsideration of the temporal framework in which channelopathies manifest.

Understanding the molecular basis opens the path toward targeted therapy. The fact that prenatal seizures are primarily associated with GoF mechanisms makes sodium channel blockers—such as phenytoin, carbamazepine, or more selective agents—a pathogenetically sound therapeutic choice. However, the main challenge and opportunity lie in prenatal intervention. Studies in animal models [112,113] have already demonstrated the feasibility of using antisense oligonucleotides (ASOs) to allele-specifically suppress the expression of mutant *SCN2A* and *SCN8A* alleles. Although this approach is still in its early stages, it points toward a future in which prenatal genetic diagnosis may enable presymptomatic or early pathogenetic treatment capable of altering the trajectory of disease development.

Future research should focus on:

1. Conducting prospective genomic studies in cohorts of infants with seizures within the first 72 h of life and/or prenatally verified seizure patterns.
2. Developing experimental models (based on iPSCs and brain organoids with identified mutations) to investigate pathogenesis and explore therapies that modify disease progression rather than merely suppress symptoms.
3. Establishing clear criteria for collecting clinical history and identifying characteristic features of prenatal epilepsy.

Only through such efforts can this “hidden” patient population be brought out of obscurity, enabling accurate diagnosis and promoting the development of effective treatment strategies based on an understanding of the underlying genetic causes.

## 5. Conclusions

Prenatal epilepsy presents a multifaceted challenge for modern medicine. Despite significant advances in perinatology and neurology, the etiology of many cases remains unclear, limiting opportunities for prevention, precise diagnosis, and effective treatment. The analysis clearly demonstrates that the key to solving this issue lies in a deeper understanding of its genetic basis. Mutations in the *SCN2A* and *SCN8A* genes represent a major cause of prenatal seizures, the pathogenesis of which extends beyond simple “neuronal hyperexcitability” and encompasses disruption of fundamental neurodevelopmental processes such as synaptogenesis and, potentially, neuronal migration. Recognizing this fact necessitates a revision of diagnostic algorithms toward more precise genetic testing in neonates presenting with seizures within the first hours of life and a paradigm shift in research toward investigating the role of ion channels in prenatal brain development.

## Figures and Tables

**Table 1:** Candidate genes for prenatal/early-onset seizures.

Gene Names	Function	Earliest Reported Age	Phenotype/Epilepsy Type
*SCN1A*	Encodes the voltage-gated sodium channel α-subunit Nav1.1 essential for action potential initiation and neuronal excitability	Within the first 3 days of life [97]	Neonatal developmental and epileptic encephalopathy with MD and arthrogryposis (NDEEMA) [97]
*KCNQ2*	Encodes the Kv7.2 subunit of M-type voltage-gated potassium channels that regulate neuronal excitability	12 h postnatal [98] Postnatal day 1 [99]	Neonatal epileptic encephalopathy [98] Benign familial neonatal epilepsy (BFNE) [99]
*KCNQ3*	Encodes the Kv7.3 subunit of voltage-gated potassium channels that co-assemble with Kv7.2 to form functional M-channels	Postnatal day 2 [100] Within the first week of life [101]	Familial epilepsy with focal seizures and intellectual disability [100] Benign familial neonatal epilepsy (BFNE) [101]
*KCNT1*	Encodes a sodium-activated potassium channel (Slack/KNa1.1) involved in regulating neuronal firing patterns	Postnatal day 4 [102] From 1 h postnatal [103]	Nocturnal frontal lobe epilepsy and epilepsy of infancy with migrating focal seizures [102] Epilepsy of infancy with migrating focal seizures (EIMFS) [103]
*STXBP1*	Encodes Munc18-1, an essential SNARE complex for synaptic vesicle docking and neurotransmitter release	From postnatal day 1 [104]	Early-onset epileptic encephalopathy (EOEE), Ohtahara syndrome, West syndrome [104]
*ARX*	Encodes a paired-like homeobox transcription factor involved in regulating neuronal proliferation, migration and differentiation during brain development	40 min postnatal [105]	Ohtahara syndrome [105]
*SLC25A22*	Encodes a mitochondrial glutamate carrier that imports glutamate into mitochondria	From postnatal day 1 [106]	Ohtahara syndrome [106]
*SLC13A5*	Encodes the Na^+^-coupled citrate transporter (NaCT) that mediates cellular citrate uptake and links citrate availability to neuronal metabolism	Postnatal days 1–2 [107]	Neonatal onset DEE with nonmigrating clonic seizures [107]
*ALDH7A1*	Encodes antiquitin (α-aminoadipic semialdehyde dehydrogenase), an enzyme in lysine catabolism	3 h postnatal [108] Postnatal day 3 [109]	Pyridoxine-dependent epilepsy [108,109]

## Data Availability

The original contributions presented in this study are included in the article. Further inquiries can be directed to the corresponding author.
